# Supplementary material for: Complexome profiling of the Chlamydomonas psb28 mutant reveals TEF5 as an early PSII assembly factor
Source: Plant Cell. 2025 Mar 17;37(6):koaf055. doi: 10.1093/plcell/koaf055 (PMC12164590; doi:10.1093/plcell/koaf055)
Supplement: koaf055_Supplementary_Data [file koaf055_supplementary_data.zip › psb28_interactive.zip!/plots/intensity/qualityPlots/peptides/ATPVH_Cre01.g027800.t1.2.html]

### Cre01.g027800.t1.2

Trivial name: ATPVH  
SeqCoverage [median]: 12.50  
Mass: 52.97 kDa  
  
 

| Sequence | MissedCleavage | Mass [Da] | wtIntensity | muIntensity | Score |
| --- | --- | --- | --- | --- | --- |
| AGGLQVLPAVIQR | 0 | 1320.79 | 1983.80 | 14472.21 | 149.72 |
| EGIVFLSNFDKYKK | 2 | 1686.90 | 246.71 | 43.75 | 43.99 |
| IIETNRDVK | 1 | 1086.60 | 1208.61 | 5946.39 | 94.85 |
| LLGHSDVEVQK | 0 | 1223.65 | 735.85 | 7396.84 | 107.03 |
| LVTDKDLQLIR | 1 | 1312.77 | 5596.22 | 25163.45 | 91.55 |
|  |  |  | 9771.2 | 53022.6 |  |
